# Evaluating the Effectiveness of Videos for Teaching Pharmaceutical Calculations to Pharmacy Students

**DOI:** 10.3390/pharmacy12010022

**Published:** 2024-01-25

**Authors:** Heba Ghazal, Marrium Haq, Philip Crilly, Nicola Harrap, Reem Kayyali

**Affiliations:** School of Life Sciences, Pharmacy and Chemistry, Kingston University London, Kingston Upon Thames, London KT1 2EE, UK

**Keywords:** pharmaceutical calculations, videos, learning tool, pharmacy students

## Abstract

Performing pharmaceutical calculations accurately is a fundamental aspect of the pharmacy profession, ensuring treatment efficacy and patient safety. Incorporating videos in teaching can enhance visualisation and reinforce learning. The current study utilised videos to teach calculations and assessed how these affected students’ performance. Initially, pharmacy students were surveyed at one UK University to identify calculation topics they found most challenging, and then two prototype videos were created based on these topics. Feedback was gathered through a follow-up survey on these prototypes, leading to the development of five additional videos. To evaluate the impact of these videos, students were given quizzes before and after watching them. The data were analysed in Microsoft Excel and included paired *t*-tests to compare mean scores, with significance set at *p* < 0.05. The survey was completed by 98/130 (75% response rate), with 58% expressing average or low confidence in calculations. A majority (78%) believed that videos would aid their comprehension of calculation concepts. In the subsequent phase, most respondents (92%, 80/87) agreed that the prototype videos improved their understanding of the two topics, but this increase was not statistically significant. However, quiz performance evaluation revealed a significant increase in average scores. This study affirms that videos can boost students’ performance in calculations by allowing them to visualise the relevant practical scenarios.

## 1. Introduction

Pharmaceutical calculations are an integral aspect of the pharmacy profession, as errors in calculations can lead to serious and harmful consequences. Pharmacists are entrusted to ensure the safe and accurate use of medicines; therefore, numeracy skills are a fundamental component of the pharmacy curriculum. Common numerical health-related tasks range from interpreting clinical data to estimating risks, calculating medication doses, dispensing prescriptions, and understanding nutritional requirements. Nevertheless, errors have occurred for various reasons, with the three most common types being: dispensing the wrong medication, incorrect dosage, strength, or formulation; wrong dose calculation or missing drug interactions or contraindications [1]. Medication errors are three times more likely to occur in pediatric patients due to the need to calculate doses based on weight. This can involve rounding numbers, with possible errors arising in the placing of decimal points [2,3]. For instance, a morphine overdose occurred, with the dose given being 100 times more than it should have been, and similarly, an error in calculating the digoxin dose for a baby was due to a decimal point misplacement [4]. A study from a UK hospital evaluated medication-related incident reports and found that overdose and underdose errors of various magnitudes occurred, with such errors happening in pediatric wards, adult wards, as well as in the pharmacy [5]. Furthermore, a surveillance study conducted in Welsh hospitals revealed instances of overdosing, sometimes up to 1000-fold, attributed to human errors, particularly the confusion of units (milligrams, grams, or micrograms) and dose expressions in mg/kg or mg [6].

Calculation errors are not confined to pediatric populations but can also occur in medications prescribed for adults, where the dose, volume, or rate of administration needs to be calculated. A study from the Norwegian incident reporting system found that calculation errors predominately occurred in hospitals, particularly when calculating doses after dilution of intravenous solutions or when setting infusion pump parameters, with the most common medicines being opiates, including morphine and oxycodone [7,8]. Another study reported that one in five intravenous medication orders placed by emergency residents in a New York city hospital deviated by more than 10% of recommended dosing [9]. These incidents reinforce the significance of calculation competency among healthcare professionals in ensuring the administration of correct doses and act as reminders of the severity of the consequences that can result from even a minor calculation error. Drug concentrations in solution can be expressed in numerous ways, such as a percentage (%), mass per volume (mg/mL), or dilution ratios (1 in x). Medications used in resuscitation and acute medical emergencies are often labelled in a ratio manner, such as adrenaline 1:1000. The struggle in converting between these expressions, as demonstrated in a study involving 150 junior doctors, in which half did not perform the conversion correctly, can increase the risk of prescribing and administration errors [10].

Calculations are based on mathematics, which is mainly an objective field. Traditional methods of teaching mathematics typically occur in a classroom setting. However, with the advancement of modern technology and the transition to a post-pandemic era, many traditional teaching methods are evolving to incorporate digital tools and blended approaches. An online package (e-package) containing calculation questions covering various pharmaceutical calculation topics was created by a UK University and subsequently, evaluated. It was found that most of the students (79%) provided positive feedback regarding the e-package impact on their calculation proficiency and nearly three-quarters (74%) were enthusiastic about its design [11]. Another study was conducted on a group of first-year university students from various courses over three weeks where students used a series of problem-based video podcasts covering major areas of mathematics as self-study tools [12]. The findings revealed that most of the students regularly utilised these video podcasts, assessed them as useful or very useful, saw them as simple, effective learning tools, and reported considerable calculus knowledge improvements. Videos aided in their understanding of the concepts being addressed, and students stated that online videos allowed them to have better control over their learning. This is supported by Maher et al. (2020) [13] who found that the use of videos in teaching was advantageous in supporting learning. Videos were also used to provide feedback to students undertaking a pharmaceutical calculations course. The students attributed the positive impact of the videos to factors such as “it can be played many times”, as this allowed them to watch and learn from these videos at their own pace compared to face-to-face teaching [14]. Thus, relevant studies have agreed that the integration of visual tools including video, pictures, and animation within teaching materials can enhance the interest in learning [15,16,17].

The Master of Pharmacy (MPharm) course in the UK typically comprises four years, followed by a mandatory one-year foundation training. Subsequently, trainees undergo an assessment set by the General Pharmaceutical Council (GPhC), the regulatory body in the UK. The GPhC deems the demonstration of calculation competency as an essential element to ensure patient safety. Based on the Miller triangle model that categorizes the levels of clinical competence as “knows”, “knows how”, “shows how”, and “does”, the GPhC requires calculation competency to be at the ‘does’ level. This signifies that pharmacy students should possess the ability to independently perform calculations in real-life scenarios, ensuring consistently reliable outcomes [18,19]. As calculations are essential for the students to progress in the MPharm course and register with the GPhC as pharmacists, teaching and assessments on calculations are embedded throughout the four years of the MPharm curriculum. Accordingly, pharmacy courses aim to explore and invest in various teaching methods and tools to equip their students to meet this competency at the “does” level [20]. While videos have been suggested as a valuable tool for teaching, there is a need to identify students’ specific topics of interest and preferred design to create videos that cater to their needs and capture their interest.

The aim of the current study is to evaluate the usefulness of short videos for teaching pharmaceutical calculations among pharmacy students and to determine the impact of these videos on their performance. The objectives were to identify the types of pharmaceutical calculations that students find most challenging, determine the features they would deem useful in a calculation video, create videos, and evaluate students’ perceptions of the videos in terms of usability and design features, and assess the impact of these videos on their perceived confidence, understanding, and performance.

## 2. Methods

### 2.1. Study Setting

The present study was conducted among the Master of Pharmacy students (MPharm) at a University in London, UK. Students were invited to voluntarily participate in the study, which took place in a university classroom with the main researcher leading the session. The study was carried out over four months.

### 2.2. Study Population

All third year MPharm students at the University were invited to participate in this study. In phase one and phase two, only third-year students participated. However, in the final evaluation phase (phase 3), recruiting the same cohort of third-year students was challenging due to their additional other course commitments and time constraints of the project; therefore, a general invitation was extended to all pharmacy students across various levels in the same university.

### 2.3. Study Design

This study was conducted in three phases. The first phase, ‘needs assessment’, used a questionnaire to identify the calculation topics that students most struggled with. Building on the findings from phase one, the second stage comprised producing two prototype videos. The videos showed a scenario with script on screen and audio reading explaining the scenario. Then, practical steps to solve the question were demonstrated. This was followed by a survey evaluating the effectiveness of these two videos. In the third phase, five new videos were designed and filmed. Students were invited to a session where they answered a quiz, watched the videos, attempted a similar quiz, and finally filled in a survey. Paper copies of questionnaires and quizzes were utilised as data collection tools, and students were also provided with a participation information sheet.

#### 2.3.1. Phase 1: Initial Questionnaire Design

The initial questionnaire consisted of 20 questions divided into three sections. Section A (5 questions) collected information on students’ confidence in calculations and their satisfaction with current methods of calculation teaching, using a five-point Likert scale. They were asked about methods they used to learn in general and methods to learn calculations. Additionally, they were requested to order different calculation topics on a scale from 1 to 10 in terms of difficulty. Section B (12 questions) inquired whether students used videos for learning in general and, more specifically, for learning calculations. A five-point Likert question was employed to identify whether they believed videos for calculations would be helpful and if they were likely to watch these videos. Students were also asked about their preferences for video design, including audio use, presenter visibility, subtitles, music background, and the preferred duration of the video. An open question sought information on other calculation topics students prefer to have videos for, and finally, Section C (3 questions) was for collecting demographic information.

#### 2.3.2. Phase 2: Prototype Videos and Their Evaluation

The feedback collected in phase one was used to design prototype videos in phase two. The results showed that displacement volumes and dilutions were ranked as the two most challenging calculation topics. Therefore, prototype videos were made on these topics, edited using iMovie, and kept short to align with the preferences expressed in the feedback. The prototype videos explained a scenario involving a calculation and, using a script onscreen with audio narration, demonstrated practical steps to solve the question. Two weeks after phase one, the trial videos were played in a teaching classroom at the end of a teaching session.

An evaluation questionnaire consisting of 21 questions was distributed after watching these videos. The questionnaire encompassed four sections: Section 1 (5 questions) gathered overall feedback by asking participants to rate the usefulness and quality of the prototype videos using a five-point Likert scale. Following this, they were queried on their interest in having more videos covering different calculation topics and their likelihood of using these videos in the future. An open-ended question sought their input on what they liked most about these videos. Section 2 (5 questions) sought views on the videos by rating their understanding and confidence levels before and after watching the two videos, displacement and dilution, using a five-point scale. Section 3 (8 questions) evaluated the quality of the videos and the clarity of concept explanations. An open question invited suggestions for any improvements, and finally, Section 4 (3 questions) asked about demographic information.

#### 2.3.3. Phase 3: Final Videos Performance and Evaluation

**Figure 1:**
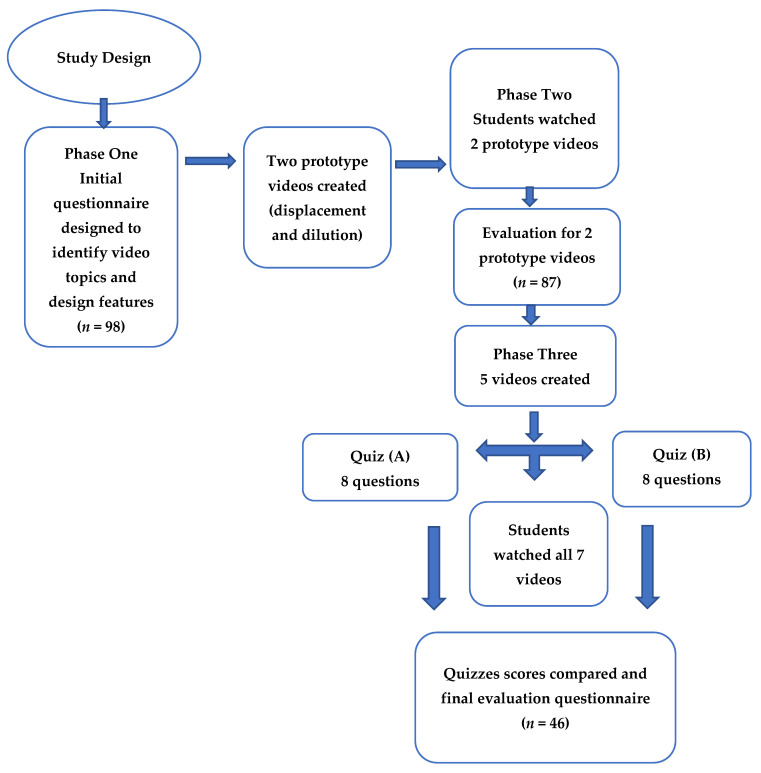
Flowchart for the study design and steps involved.

A further five videos were designed and created, covering the following five topics, which students indicated in phase one that they also struggled with: alligation, concentrated waters, concentrations, infusion rate, and molarity. The design of the prototype videos received positive feedback in phase two but asked for the inclusion of written text explanations of the calculation; consequently, the design was retained in these five videos with explanations added. Pharmacy students from various years were invited to a workshop-style session in a teaching classroom, which took place eight weeks after phase two. They were asked to solve quiz A (baseline quiz), which consisted of 8 open-ended questions to be completed in 16 min. The topics tested encompassed all the seven mentioned topics, with two questions specifically focused on molarity. After the completion of quiz A, participants watched all seven calculation videos (two prototype videos and the five new videos), one after the other. They then completed quiz B (similar to quiz A) in 16 min to determine if their scores improved from the baseline. Following quiz B, students were given an evaluation questionnaire comprising 20 questions similar to the questionnaire used in phase 2. The questions covered topics such as the usefulness of the videos, confidence, understanding, and evaluation of video quality. The full study design is illustrated in Figure 1.

### 2.4. The Videos

The videos were designed and recorded by fourth-year pharmacy students within the pharmacy practice suite at the University. Each video started with a calculation problem, followed by a practical demonstration of the process and an explanation of how the question could be solved. The average duration of the videos was 2.4 min. The videos were recorded using the researcher’s personal device and edited using iMovie on an iMac computer. iMovie is a video editing application that allows text addition and trimming within a clip. For instance, in the displacement video, an amoxicillin 125 mg/5 mL bottle was displayed, with the researcher highlighting the reconstitution instructions on the label and zooming in on the details that specify adding 86 mL of potable water to make a 100 mL of amoxicillin suspension. Then, the suspension was prepared, showing a final volume of 100 mL produced using a conical measure, followed by explanations of the steps required to calculate the displacement volume of 125 mg of amoxicillin.

### 2.5. Data Analysis

The study population in both phase one and two consisted solely of third-year Mpharm students, which, as per university records, is *N* = 130. Based on this figure and using the Raosoft^®^ (2004) sample size calculator with a confidence level of 95% and a margin of error of 5%, a minimum recommended sample size of 98 was calculated [21]. However, in phase 3, a general invitation was extended to pharmacy students. Accordingly, Raosoft^®^ sample size calculator was not applied.

All survey responses were inputted into Microsoft Excel. Descriptive statistics, including percentages, frequencies, means, and modes were used. Weighted average Likert scores were calculated for before and after watching the videos. The Chi-square test was employed to examine the association between gender and finding the videos useful at a significance level of 0.05. The average score was calculated for each quiz. A two-tailed, paired *t*-test was also conducted to determine whether the differences between student scores in quiz A and quiz B (before and after watching the videos) were significant, indicated by a *p*-value of less than 0.05. The open-ended questions were analysed using thematic analysis.

### 2.6. Ethics

This study received ethical approval before commencing any data collection from the delegated ethical approval team operating under the ethics committee of the University’s Science, Engineering, and Computing Faculty. Ahead of taking part in the study, paper copies of participation information sheets were handed to the participants. Participation was considered as implied consent, and students were advised that they were under no obligation to participate and could withdraw up until the point of final submission.

## 3. Results

### 3.1. Phase 1: The Initial Survey

There were a total of 98 participants in phase one, which matched the calculated sample size in Section 2.5, resulting in a response rate of 75% (98/130). Most participants were female (69%, 68/98) and 28% (27/98) were male.

Most students were in the 18–25 years age category (94%, 92/98), and the largest ethnic backgrounds were Asian/Asian British (52%, 51/98) and Black/African/Black British (17%, 17/98). The majority of students (58%, 57/98) rated their confidence with calculations as average, low, or extremely low, while (42%, 41/98) rated their confidence positively as confident or extremely confident.

The preferred method of learning calculations was attending workshops (70%, 69/98), followed by revision using past paper exams (63%, 62/98), and students’ university online platform questions, “calculation e-package” (48%, 47/98). Videos were selected by 32% (31/98) of the participants. Furthermore, a majority (79%, 77/98) expressed a willingness to use videos that explained calculations.

When students ordered 10 pharmaceutical calculation topics in terms of difficulty, displacement values and displacement volumes were identified as the most challenging topics followed by infusion rates and alligation. Table 1 shows the topics students preferred to be included in the designed videos.

Regarding video design features, 89% (87/98) favoured videos that included both visual and audio elements, as opposed to those without an audio narrative, with a preference for video length of less than 10 min (79% (*n* = 73/92)). Accordingly, the designed videos were kept short. Most participants (83%, 77/93) also indicated a preference for the presenter not to be fully visible in the video and no music background.

### 3.2. Phase 2: Prototype Videos and Their Evaluation

**Figure 2:**
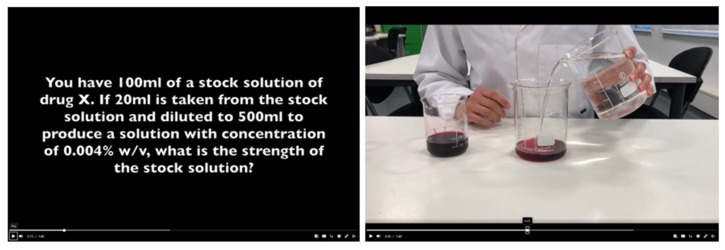
Snapshot of a video showing an example of a dilution scenario and the practical steps involved in solving the question.

Based on the results obtained in phase 1, where 79% of the participants expressed a willingness to watch videos that explained calculations, two prototype videos were created. The videos were designed to meet students’ stated preferences, with both visual and audio elements, and without a presenter visible and background music. A snapshot example of one of the created videos is shown in Figure 2.

The prototype videos were played at the end of one of the third-year MPharm teaching sessions, and an evaluation questionnaire was distributed to gain feedback on the two videos and assess the impact of these videos on students’ confidence and understanding. Out of the 100 students who were present in the session, 87 filled in the questionnaire, resulting in a response rate of 87% (87/100).

Table 2 shows the demographics of phase two participants. The majority were female (71%, 62/87), and the largest age group among students was 18–25 (92%, 80/87).

The evaluation questionnaire sought students’ views on the usefulness of the two prototype videos (*n* = 87). Most of the participants (86%, 75/87) found the videos either useful or very useful, and no significant association was observed between gender and rating the videos as useful. The Chi-square test showed *p* = 0.093 (*p*-value > 0.05). They reported they would consider using the videos again in the future, with the majority (91%, 79/87) responding affirmatively and either agreed or strongly agreed that they would appreciate having more videos covering additional calculation topics.

The clearest theme that emerged in students’ responses when answering the open question about what they liked most about the calculation videos was that it helped them to visualise the calculation scenarios (57%, 47/83) (Table 3). Other themes identified were clear explanation 13% (11/83), concise videos 11% (9/83), and a step-by-step process 11% (9/83).

The vast majority of students (91%, 79/87) found the quality of the videos to be either good or very good, and the duration to be appropriate (87%, 76/87), with ideal sound volume (97%, 84/87) and ideal speed (75%, 65/87). The explanation of the concepts in the videos was rated either good or very good 93% (81/87). Just over half of the respondents said they would prefer the videos to include a written explanation of the calculation.

**Figure 3:**
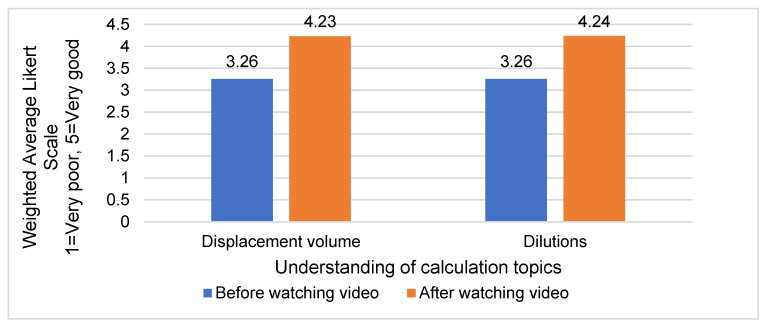
Weighted average Likert scale for students’ reported understanding before and after watching the displacement volume and dilution videos.

Students’ confidence in answering displacement volume questions, assessed using a scale ranging from 1 (extremely unconfident) to 5 (very confident), was 3.11 before watching the video and increased to 4.25 after watching the video. Although there was an increase in confidence reported after watching both the displacement volume video and dilution video, two-tailed, paired *t*-tests revealed that the differences were not statistically significant (*p*-value > 0.05). Students were also asked to rate their understanding of the two topics before and after watching the videos using a Likert scale (Figure 3). Despite an observed increase, these changes were not statistically significant (*p*-value > 0.05). Overall, most students (92%, 80/87) either agreed or strongly agreed that the videos helped them better understand the concepts of the two calculation topics, with 78% (68/87) reporting feeling more confident in the calculation topics after watching the videos.

### 3.3. Phase 3: Final Video Performance and Evaluation

As the results of phase 2 were positive, with students finding the prototypes useful and the quality of videos appropriate, a common request emerged to include a written explanation for the working out. Consequently, this was addressed, and the same videos design was maintained to create an additional five videos on the topics suggested in phase 1. In the final session, pharmacy students from different academic years were invited.

Forty-six students participated in phase three, representing various levels in the pharmacy course: 5 students from year one, 19 students from year two, and 21 students from year three. It is worth noting that the topics included in the videos were taught from the first year and upward in this MPharm course; therefore, students at various levels were expected to be familiar with these concepts. The majority indicated videos had good or very good quality (85%), and no significant association was observed between gender and finding the videos useful, as indicated by the Chi-square test with *p* = 0.94, (*p*-value > 0.05).

**Figure 4:**
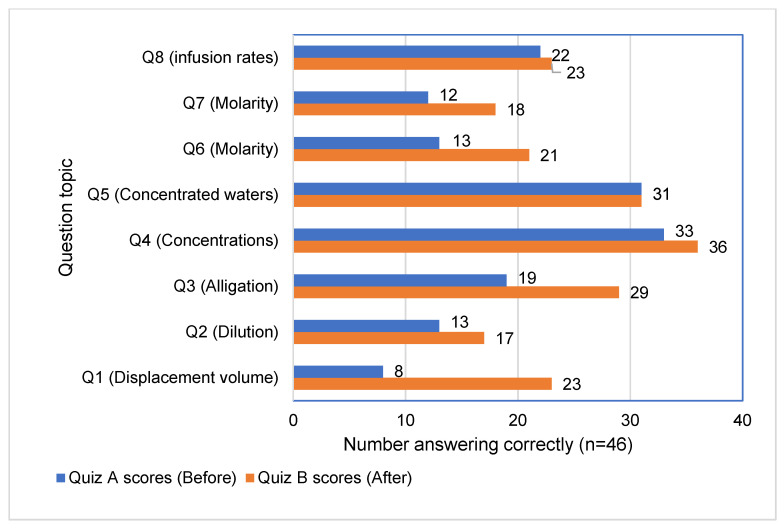
Number of participants who answered each question correctly before and after watching the videos (*n* = 46).

The participants attempted the two sets of quizzes. The average score achieved in quiz A was 3.28/8 (41%), and the average score achieved in quiz B was 4.30/8 (54%), indicating an increase in marks. After conducting a two-tailed, paired *t*-test, a *p*-value of 2.71 × 10^−5^ was obtained, showing that this difference was statistically significant (*p*-value < 0.05). In addition to the improved quiz scores, most of the participants in this phase found the videos useful and reported an increase in their understanding and confidence levels related to the topics. The majority (59%, 27/46) agreed or strongly agreed that their confidence increased, and 80% (37/46) reported improved understanding of the covered concepts. This shift in perceived understanding was reflected in the improved quiz performance. Figure 4 shows the number of participants who answered each question correctly before and after watching the videos.

## 4. Discussion

In summary, this study identified the pharmaceutical calculation topics that students found challenging and explored their perceptions of videos for learning. Videos explaining calculation topics were then created, and their impact was evaluated. The evaluation showed an improvement in students’ knowledge, as well as a suggested increase in perceived confidence and understanding after watching the videos, although this was not statistically significant.

The present study discussed the value of using videos to teach pharmacy students pharmaceutical calculations using a student-centred approach. In the initial survey, students were asked about the topics they found challenging, the types of videos that would be beneficial, and the desired quality of these videos. This approach involved students in the project from the outset. Furthermore, the videos were created by a pharmacy student, which added an extra element of peer-assisted learning and allowed students to convey their perspectives to their peers. A common theme throughout this project was the importance of visualising information through videos, closely linked to the need for experiential learning. To the best of our knowledge, no studies have adopted the same pedagogical approach in their methodology.

The finding suggests that the use of videos to teach undergraduate pharmacists can improve their performance, as demonstrated through better quiz scores before and after watching the videos. Phase one survey determined the need to expand the current teaching of calculations with a newer tool so that students are performing at the required ‘does’ level. Videos can be an effective tool for enhancing cognitive learning by transforming information into a visualised practical process combining visual, auditory, and written elements to support learning. Students have previously found videos feedback valuable due to its flexibility, allowing them to watch and review the content at a time and place that suits their pace and needs [13]. Offering accessible videos that can be revisited is more likely to enhance comprehension and retention, suggesting that videos can serve as a supplementary learning tool for calculations.

Additionally, incorporating videos into a blended learning approach can enhance students’ learning experience, which is explained by the ‘cognitive theory of multimedia learning’. This improvement was attributed to the increased ability of students to manage their cognitive load by their ability to pause and rewind [22]. This aligns with observations of previous studies that have noted enhanced performance after watching videos compared to conventional methods of teaching [23,24]. When employing video podcasts for teaching purposes, the key benefits reported were improved affective and cognitive attitudes towards the podcast and greater autonomy over learning processes [25,26]. Moreover, utilising a video assignment resulted in a higher rating of satisfaction among students and fostered increased engagement compared to the textbook assignment [27].

The use of pre-recorded videos can be a valuable tool in teaching; for instance, Cotta et al. (2016) used a flipped method for teaching pharmaceutical calculation where students were required to watch pre-recorded lectures before the class, allowing the class time to be utilised for working through problem-based scenarios [28]. The authors concluded that pre-recorded videos serve as student-centred active learning. Similar findings were reported from a study that employed a flipped classroom approach and led to better performance in pharmacy calculation skills [29]. Thus, the video developed in the current study and future recorded videos can be employed in a flipped learning approach, diverging from the typical lecture format.

Furthermore, as these videos were created by fourth-year pharmacy students, this added value by allowing the student to transfer their vision and knowledge to their peers through these videos in a relatable manner. The utilisation of peer-assisted learning (PAL) in clinical teaching to pharmacy students had shown evidence that it can improve students’ learning [30], and the present study can be counted as an implementation of the PAL concept.

This study encountered some limitations. Firstly, the final evaluation phase had a low level of participation from third-year students, attributed to their other course commitment and time constraints related to this project. Therefore, to seek wider views on the created videos, students from various academic levels in the pharmacy course at the same university were invited to phase three. The topics covered in the video are taught in the first year and upwards in this MPharm course, making the inclusion of participants from various levels beneficial. Nevertheless, there were uncontrolled variables in terms of students’ level and knowledge even within the same year level. Therefore, it was challenging to control the differences in numeracy skills and background knowledge among the participants in the sessions. An interesting extension of this work could involve streaming students based on their academic level and mathematical abilities followed by conducting the survey to determine whether our findings are correlated with students’ levels and knowledge. Secondly, the performance evaluation, including watching videos and quizzes, was carried out in the same session. It would have been more beneficial if the study was spread over a longer period to assess the impact and retention of the knowledge acquired.

## 5. Conclusions

Teaching pharmaceutical calculations can take various formats, and the current study suggests videos as one of the effective tools. The use of videos to illustrate pharmaceutical calculations showed a positive impact on student performance, and it is recommended as an accessible and supplementary resource to help students visualise the calculation scenarios and thereby enhance their performance in this area.

## Figures and Tables

**Table 1:** Top five pharmaceutical calculation topics students wanted to be included in the videos (75 responses).

Calculation Topics	Number of Students (%) (*n* = 75)
Displacement volume	24 (32%)
Displacement value	22 (29%)
Alligation	22 (29%)
Dilutions	16 (21%)
Infusion rate	15 (20%)

**Table 2:** Demographic characteristics of respondents in phase two (*n* = 87).

Characteristics	Number of Participants (*n* = 87) *n* (%)
Gender	
Male	25 (29)
Female	62 (71)
Prefer not to say	0 (0)
Age	
18–21	39 (45)
22–25	41 (47)
26–30	2 (2)
31–35	1 (1)
36–40	3 (3)
Over 40	1 (1)
Ethnicity	
White	9 (10)
Asian/Asian British	47 (54)
Black/African/Caribbean/Black British	16 (18)
Mixed Ethnic Group	5 (6)
Prefer not to say	5 (6)
Other	5 (6)

**Table 3:** Thematic analysis of why participants liked the videos.

Theme	Illustrative Quotes
Visualise the scenario	‘Visualising the calculation’ ‘The visual representation of the question’ ‘The videos made it easy to visualise what is happening in a calculation’ ‘It helps to visualise the question’.
Clear explanation	‘The calculation steps are clear’ ‘The videos were clear’ ‘The visual explanation of complicated concepts’.
Step-by-step process	‘I like the step by step process’ ‘step by step calculation with good explanations’ ‘Explanation of each step’.
Videos were concise	‘I understand it. Simple and short’ ‘Short, to the point’. ‘They were of a short length and succinct in nature, concise’.

## Data Availability

Data are contained within the article.
